# A novel body mass index reference range - an observational study

**DOI:** 10.6061/clinics/2017(11)09

**Published:** 2017-11

**Authors:** Sirlei Siani Morais, Mirena Ide, Andrea Moreno Morgan, Fernanda Garanhani Surita

**Affiliations:** Departamento de Ginecologia e Obstetricia, Faculdade de Ciencias Medicas, Universidade de Campinas (UNICAMP), Campinas, SP, BR

**Keywords:** Gestational Weight Gain, Body Mass Index, Prenatal Care

## Abstract

**OBJECTIVE:**

To generate a new body mass index curve of reference values and ranges for body mass index and weight gain during pregnancy and to compare the new curve and weight gain ranges with the currently used references.

**METHODS:**

A prospective observational study was conducted with a total of 5,656 weight and body mass index measurements in 641 women with single pregnancy who attended their first prenatal visit before 12 weeks. All the women were over 18 years old and had no medical conditions that would influence body mass index. Data were collected using prenatal charts and medical records during hospitalization for childbirth. A linear regression method was used for standard curve smoothing in the general population and for specific curves according to the baseline body mass index classification. Curves were obtained for the 5^th^, 10^th^, 50^th^, 85^th^, 90^th^ and 95^th^ percentiles. Concordance between the classification of women using the newly generated and currently used curves was evaluated by percentages and kappa coefficients. The weight gain was compared with the reference values of the Institute of Medicine using Student’s T test. The data were analyzed using SAS software version 9.2, and the significance level was set at 5%.

**RESULTS:**

A general reference curve of percentiles of body mass index by gestational age was established. Additionally, four specific curves were generated according to the four baseline body mass index categories. The new general curve offered percentile limits for women according to their initial body mass index and according to the Centers for Disease Control and Prevention limits, showing poor agreement with the currently used curve (48.3%). Women who were overweight or obese when starting prenatal care had higher weight gain than the Institute of Medicine recommendation.

**CONCLUSIONS:**

The new proposed curve for body mass index during pregnancy showed weak agreement with the currently used curve. The new curve provided more information regarding body mass index increase using percentiles for general and specific groups of body mass index. Overweight pregnant women showed an upward body mass index trend throughout pregnancy that increased more dramatically than those of other groups of pregnant women, and they also presented a major mean difference between weight gain and the Institute of Medicine recommendation.

## INTRODUCTION

Nutritional disorders have become a worldwide problem due to the high prevalence of obesity during different stages of life. For women of reproductive age, two extreme conditions should be considered: on the one hand, obesity and excessive gestational weight gain (WG), and on the other hand, women with insufficient WG who compulsively avoid increased weight during pregnancy 1-6.

Several recommendations for gestational WG have been adopted in different populations based on different parameters, such as weight or body mass index (BMI) before pregnancy and distribution curves of increased BMI or WG ranges during pregnancy. However, because of intense socio-cultural and behavioral changes in the last few decades, it is necessary to update the scientific knowledge and the normal range for these recommendations and, therefore, to establish parameters for health professionals to guide pregnant women 7,8.

These recommendations differ among countries. In Sweden, Germany, Switzerland, Austria and Turkey, the recommendations are based on caloric intake. The Institute of Medicine (IOM) in the United States suggests a weekly WG range by trimester based on pre-pregnancy BMI. WG recommendations according to pre-pregnancy BMI are followed by other countries (Italy, Vietnam, Western European countries, Australia and Pacific Islands). The total WG forecast is also used in clinical practice. Increased caloric intake until the end of pregnancy is recommended in Japan. Singapore uses WG according to height, and the Philippines, India and Sudan use a recommendation of simply gaining weight and following a good diet 7.

In Brazil, the Ministry of Health suggests the use of Atalah’s curve 9, which incorporates the intersection of BMI and gestational age and has the advantage of not requiring a standard reference. BMI use simplifies nutritional assessments during pregnancy, is easy to calculate and demonstrates a good association with the degree of adiposity and the risk of non-communicable chronic diseases 9,10.

However, the use of Atalah’s curve in Brazil should consider the pre-pregnancy BMI classification, which differs from the current WHO classification. The curve was developed a few decades ago; it does not provide clinical parameters to evaluate upper and lower limits for obese and underweight women and was developed from a cross-sectional study of Chilean women 7,9,11.

The purpose of the present study was to assess BMI during pregnancy using a longitudinal study of Brazilian pregnant women to generate a reference curve according to pre-pregnancy BMI and, therefore, to provide new parameters that could be used to monitor the weight of women during pregnancy.

## MATERIALS AND METHODS

The research protocol for this study was approved by the Institutional Review Board of the School of Medical Sciences of the University of Campinas and took into account all the requirements established by the Brazilian National Health Council.

A prospective observational study was conducted at the State Hospital of Sumaré (SHS), a university teaching hospital that is a reference for low-risk pregnant women and is affiliated with the University of Campinas. This hospital is located in the metropolitan region of Campinas, state of São Paulo, Brazil. The state of São Paulo has the highest population density in the country, with an estimated population of 41 million, and the metropolitan region of Campinas has the third largest population of women of reproductive age 12,13.

From March to October 2015, data were collected from prenatal charts and the medical records of women who gave birth at the SHS. Women who had some conditions that could influence BMI during pregnancy, such as diabetes, drug use, HIV infection, multiple pregnancy or cancer, as well as women without prenatal care, with onset of prenatal care after 16 weeks of gestational age or women under 18 years of age, were excluded.

The sample size was calculated to evaluate the BMI variation with a representative number of weight measurements in all gestational ages. We used a reported BMI mean value of 24.2±4.5 kg/m^2^ in pregnancy. Considering a significance level of 5% and variation of 2%, the sample size was estimated to be 333 women (to evaluate the BMI variation) 14. To evaluate the measurement, it was estimated that most women had approximately six prenatal visits during pregnancy. According to data from SHS in 2009, for the 2,340 deliveries that occurred there, an average number of 14,040 measurements would be available 15. Considering a significance level of 5% and a sampling error of 2%, the sample size was calculated as 2,050 measurements. A total of 849 clinical records were assessed, among which 753 met the inclusion criteria and 641 contained complete information as required by the study protocol. The records showed that the prenatal charts contained between 6-16 weight measurements, resulting in 5,656 weight measurements at different gestational ages.

The inclusion criteria were verified with a checklist, and the available data were transcribed into a specific form and stored in an Excel file. Double entry of the data was performed, and then the data were validated in Excel. After a detailed consistency checking procedure, inconsistencies in the database were reassessed using the data collection form, clinical records and prenatal charts as the main sources of data.

### Data analyses

For maternal and perinatal data assessments, absolute and relative frequencies were used for the sample of 641 women. They were classified according to the first BMI evaluation during prenatal care, as defined by the WHO criteria using the weight/height^2^ formula, into four categories: low weight (<18.5 kg/m^2^), adequate weight (18.5 to <25.0 kg/m^2^), overweight (25.0 to <30 kg/m^2^) and obese (≥30 kg/m^2^) 16.

The weight measurement was first assigned using the dependent data; however, because the measurements were collected from prenatal charts, there were substantial missing data at many gestational ages. Therefore, the values were studied in independent form: each gestational age and weight was considered one measurement of the sampling unit. All the measurements and a stratification of the sample according the classes of initial BMI (WHO criteria) were evaluated to determine the equation to describe the evaluation of the change in weight and BMI according to gestational age during pregnancy using a simple linear regression, and they were found to be normally distributed. However, the coefficient determination (R^2^) estimated for the curve by linear regression was low, and the curves were considered to have low predictive value.

The 5^th^, 10^th^, 50^th^, 85^th^, 90^th^ and 95^th^ percentiles for gestational age for the entire sample and for initial BMI stratification were described. A simple linear regression was also used for these new values to smooth the curve, and the equation was used to estimate the reference values for each percentile. The smoothed equation of the percentiles that was obtained from the entire sample curve was then used to classify each of the 641 women during early pregnancy (first measure), in the middle of pregnancy (between 19-23 weeks) and at the last prenatal evaluation (between 35 to 41 weeks). We classified the women according to four categories using the same percentiles as the Centers for Disease Control and Prevention (CDC) 17: low weight (those with BMI <P5), adequate weight (BMI ≥P5 and BMI <P85), overweight (BMI ≥P85 and BMI <P95) and obese (BMI >P95).

The mean WG during pregnancy was then compared with the IOM recommendation, and the difference was calculated and compared using Student’s T test.

The women were also classified by their BMI using two different instruments, the newly proposed curve and Atalah’s curve, during the three different periods of pregnancy: early, middle and late. The classification by Atalah’s curve was then compared with the classification by the new curve to assess prominent modifications. McNemar’s test and the weighted kappa coefficient, with its respective confidence intervals, were used. The significance level was 5%, and the data were analyzed using SAS version 9.4 software Copyright (c) 2002-2012 by SAS Institute Inc., Cary, NC, USA.

All STROBE statement items for a prospective study were followed and checked in this manuscript 18. Financial support for the current study was obtained from the São Paulo Research Foundation (FAPESP), grant number 2014/01770-7. The content of this article is solely the responsibility of the authors and does not necessarily represent the official views of FAPESP, which did not influence the content of the manuscript.

## RESULTS

A total of 5,656 weight and BMI measurements from 641 women with single pregnancy and first prenatal visit before 16 weeks were obtained. Most of the women had white skin color (62%), were 20-34 years old (76.5%), and were primiparas (74.5%), and maternal anemia was present in 15.3% of the sample. Prenatal care was started between 6 and 12 weeks in 72.9% and between 12 and 16 weeks in 23.6% of the women.

The first BMI measurement during prenatal care classified 47.1% of the women as adequate weight, 30.9% as overweight, and 16.7% as obese, resulting in a total of 47.5% of women with excessive weight. Vaginal delivery occurred in 59.8% of all pregnancies. Neonatal results showed that most newborns weighed 2,500-4,000 g (86.7%). Macrosomia occurred in 5.1% of newborns. Complete data with some characteristics of the sample are presented in Table 1.

The mean increase in BMI during gestation was 2.7 to 4.6 units, representing a percentage mean increase in BMI between 8.2 and 26.1%. Of the women classified as obese at the first prenatal visit, 33.7% had excessive gestational WG according to the IOM recommendation (Table 2).

Women classified at the first prenatal visit as low weight or adequate weight did not present any significant difference between their gestational WG during pregnancy and the IOM recommendation. Women classified as overweight at the first prenatal visit had a mean WG of 4.1 kg above that recommended by the IOM, and those classified as obese had a mean WG of 2.2 kg above the IOM recommendation.

Using the equations presented in Table 3, we can estimate the percentiles of BMI for any gestational age.

However, we estimated the 5^th^, 10^th^, 50^th^, 85^th^, 90^th^ and 95^th^ percentiles for each gestational age to evaluate the BMI of the pregnant women using the equation for each gestational age between 8 and 40 weeks. The reference values for these percentiles are available in Table 4 or were alternatively generated using an Excel file calculator provided by the authors at https://www.dropbox.com/s/mbsfldtv257n6zn/calculadora%20-%20link.xlsx?dl=0.

**Figure 1:**
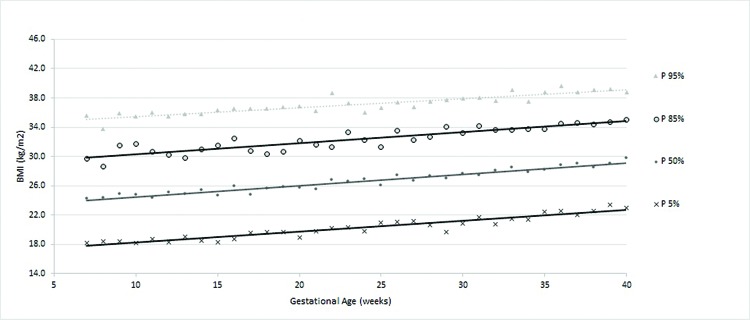
Percentiles and smoothed curves of BMI by gestational age – total sample (any initial BMI).

**Figure 2:**
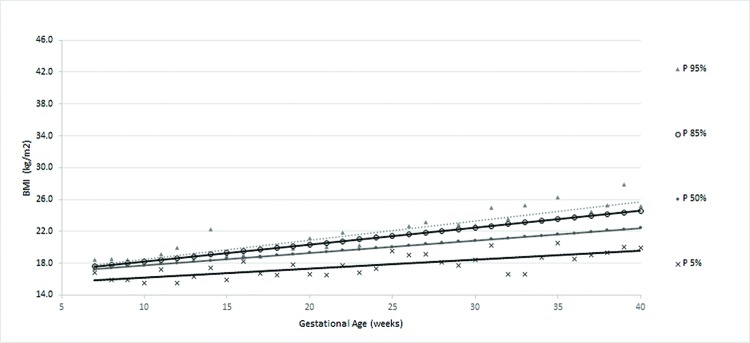
Percentiles and smoothed curves of BMI by gestational age for women classified as low weight at the first prenatal visit.

**Figure 3:**
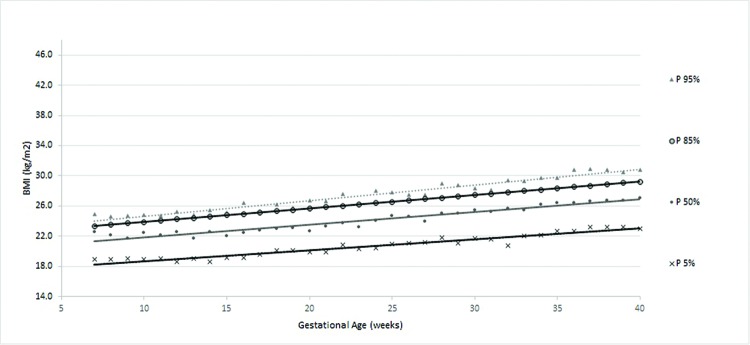
Percentiles and smoothed curves of BMI by gestational age for women classified as adequate weight at the first prenatal visit.

**Figure 4:**
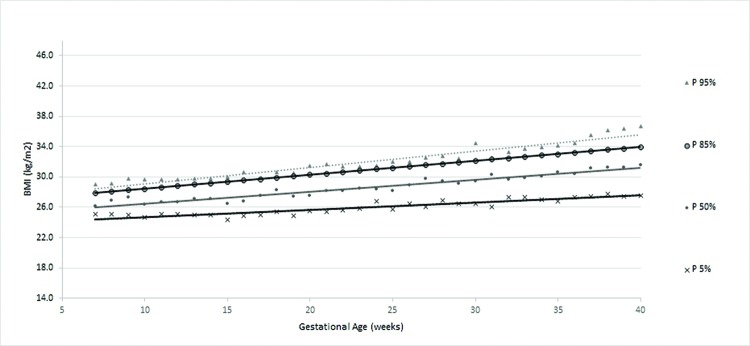
Percentiles and smoothed curves of BMI by gestational age for women classified as overweight at the first prenatal visit.

**Figure 5:**
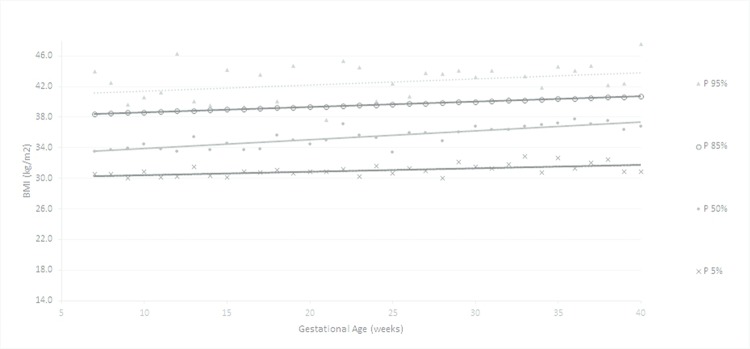
Percentiles and smoothed curves of BMI by gestational age for women classified as obese at the first prenatal visit.

According to the obtained reference value data, a general curve was created. Four other specific curves were generated according pregestational BMI to monitor WG and consequent changes in BMI during pregnancy (figures 1 to 5).

The ranges of BMI values between percentiles 50 and 90 or between 50 and 95 were larger than the ranges of BMI between percentiles 5 and 50 or 10 and 50, showing a trend toward a higher concentration of cases in the upper part of the curve.

The four smoothed curves generated according to the first prenatal BMI were similar to the general curve at some points. However, there were different slopes by initial BMI. The slope of the line generated for the 50^th^ percentile was higher among women with a normal weight (slope=0.167), followed by overweight (slope=0.159), low-weight (slope=0.156) and obese women (slope=0.115), demonstrating that the majority of women (50%) who initially gained weight had an adequate weight or were overweight (Table 3).

The curve of the women with low weight (figure 2) displayed a greater dispersion of BMI values during the final weeks than in the first week of prenatal care. The mean WG in these women with low weight was 12.1±4.7 kg, ranging between 0.80 kg and 24.9 kg. The average percentage of WG was 26.1±10.6%, ranging from 1.6% to 51.7% (data not shown).

In women with an adequate initial BMI, the new curve (figure 3) showed that BMI increases occurred proportionally throughout gestation, as shown by the parallel percentile lines. The average WG of women with an adequate BMI was 11.5±5.0 kg. The mean percentage of WG was 20.3±9.0% (data not shown).

For women classified as overweight, the slopes of the percentiles were mostly lower than those of the low-weight, adequate-weight and obese women (figure 4). The average WG (in kg) of overweight women was 10.1±6.4 kg, ranging from -10.6 kg to 28.2 kg. The average percentage of WG was 14.5%±9.5% (data not shown).

Obese women displayed a curve with a broad slope (figure 5), but an increase in data dispersion (variation of percentiles around the smoothed line) and a greater range were observed compared with those in the other groups. The average WG percentage was 7.2%±5.8%, ranging from -9.4% to 28.8% (data not shown).

The agreement between the new curve and Atalah’s curve was approximately 50%. Among the 51.7% of women with discordant data in early pregnancy, 39.8% (27.7% overweight/adequate + 12.1% obese/overweight) represented an underestimation of Atalah’s curve compared with the new curve for women classified as underweight or overweight by Atalah’s curve. In the middle of pregnancy, the most discordant findings (25.8%) were for women classified as overweight by Atalah’s curve and adequate by the new curve. At the end of the prenatal period, this percentage was 26.7%. There was complete disagreement regarding women classified as overweight by Atalah’s curve. In general, Atalah’s curve provided classifications with good agreement for adequate women but with disagreement for low-weight and overweight women. The agreement could be considered weak for all the evaluated data based on the values obtained by the weighted kappa (Table 5).

## DISCUSSION

The results of this study add reference values for BMI throughout gestation, both with regard to the lower limit through the 5^th^ and 10^th^ percentile curves and to the upper limits through the 90^th^ and 95^th^ percentile. The smoothed curves provide health teams with clinical tools to evaluate the BMI of pregnant women at each gestational age throughout pregnancy. A pregnant woman’s BMI values can be evaluated using the curve of the general population and, more specifically, through the different curves established according to BMI in early pregnancy using the WHO criteria (four categories). The standard increase in BMI during pregnancy was specific to each classification of early BMI in prenatal care, as observed in other studies 11,19,20.

The new curves showed weak agreement with the current curve (Atalah’s). However, an overestimation of the values at the beginning of prenatal care was observed, which is consistent with previous comparisons with Atalah’s curve and is likely due to ethnic and cultural differences between Atalah’s original population and the Brazilian sample 21,22. Although it is the standard and recommended by the Ministry of Health in Brazil, Atalah’s curve has limitations, such as a lack of upper limits for pregnant women classified as obese and lower limits for pregnant women classified as underweight 9,12. Atalah’s curve was developed in the 1990s; since then, there have been changes in behavior and gestational weight recommendations, as well as cultural and racial influences that might impact the results in different countries 16,23.

The results were based on a specific population with certain socio-demographic and cultural characteristics, which could be considered a standard low-risk population of pregnant women. These women had the expected nutritional status for a middle-income setting, a low rate of anemia and a proportion of overweight women similar to those previously described. To be applied at a national or higher level, these results would need to be validated with data from larger samples and from different populations. This method could minimize the influence of factors such as ethnicity, nutritional status or educational level. Hopefully, the present study population could be considered a representative sample of Brazilian pregnant women providing results that should be considered for public health purposes 12,24.

The sample size was estimated for the total sample, not for a stratified sample (using the initial BMI such as presented in this article). In this case, an increase in the variability of the small stratum occurs, such as the women classified as low weight at the beginning of prenatal care. At most gestational ages, there were fewer than 10 observations, and in these cases, the rank statistics (such as percentiles) were strongly affected by the sample size 25. These results must be used and evaluated with some restrictions, since a limitation of this study is the altered precision due to the reduced stratified sample size.

Another limitation of this study can be considered the statistical method. The approach that was applied was an empirical method followed by a regression analysis to smooth the curves. The absence of dependency of the data between measurements may have resulted in a loss of variance (total and specific models) because we did not consider correlations. However, the number of samples that could be analyzed with dependency was very small due to the lack of data, and the global variance tended to be larger because of the small sample size. Additionally, Atalah’s curve (actual reference curve) was not generated using dependent data 9,25.

The curves displayed approximately similar shapes with different levels that were identified when the data were stratified according to the four possible categories of BMI during early prenatal care. Women with low weight early in pregnancy gained weight faster in late pregnancy, as evidenced by the small range of the smoothed percentiles. Women with an adequate initial weight exhibited a uniform BMI increase throughout pregnancy, as demonstrated by the straight and parallel lines with a small range between percentiles at different gestational ages. Other studies have also shown that the differentiation of BMI categories provides important insights into neonatal and maternal outcomes 26,27.

The evaluation of curves for overweight women suggests that this group requires special attention because they have a greater range of percentiles during late pregnancy and a steeper slope (sharper increase), which was also observed for underweight women. Overweight women displayed a similar WG to that of women with an adequate WG and a greater WG than that of obese women. Obesity is a known risk factor for adverse outcomes during pregnancy, often representing a bias because obesity may induce clinicians to underestimate the WG problem in overweight and/or adequate-weight women 27.

When the IOM guidelines are considered, excessive WG is again highlighted in the overweight group; similar data have also been described in other studies 28,29. These results demonstrate the need for special attention in overweight women.

It is natural to anticipate that a standard curve according to the nutritional profile of each woman at the beginning of pregnancy would be feasible and useful. The current proposed model presents BMI percentile curves for women in general and according to BMI at early prenatal care. The results suggest a need for important changes in how BMI and WG are recommended and monitored during pregnancy 26,27. It would be possible and easier to follow the increase in percentiles of BMI similarly as for weight and height increases in children.

Guidelines are extremely important to support health care professionals in treating pregnant women and newborns, but we must consider the difficulties inherent in personal care, such as personal motivations and psychological support, that mathematical curves do not reflect. Thus, using curves is a simple method to professionally assess gestational WG in an individualized way. In Brazil specifically, this practice has been part of the prenatal routine for many years; our suggestion is that the currently used curve be replaced by the new proposed curve because it was created for Brazilian women.

Therefore, a BMI classification of a pregnant woman can be provided according to gestational age, and assessments of the changes in her BMI can be compared to standard percentiles. These new curves seem to be useful and provide another tool for health professionals to monitor maternal health. In addition, depending on the provider’s decision, they can follow only women who are in the upper or lower limits for WG 11,23,30.

The new curves developed in this work showed weak agreement with Atalah’s curve and provided additional information regarding BMI growth percentiles for general and specific body compositions. Women classified as overweight at the first prenatal visit had higher than recommended WG and an upward trend of BMI throughout pregnancy that was sharper than for the majority of pregnant women, indicating the need for a special focus on overweight women beginning in early prenatal care.

## AUTHOR CONTRIBUTIONS

The idea for the study and this specific analytical approach were developed by Morais SS and Surita FG. Analyses were planned and performed by Morais and Surita FG. The first version of the manuscript was drafted by Morais SS and Surita FG and then complemented with suggestions by Ide M and Morgan AM. Morais SS, Surita FG, Ide M and Morgan AM contributed to the development of the study protocol and approved the final version of the manuscript.

## Figures and Tables

**Table 1:** Sociodemographic, nutritional, delivery and neonatal characteristics of the sample.

Maternal characteristics	(n = 641)	%	Delivery and neonatal characteristics	(n = 641)	%
**Ethnicity (n = 619)**			**Mode of delivery (n = 634)**		
White	396	62.0	C-section	263	40.2
Non-White	243	38.0	Vaginal	391	59.8
**Age (n = 639)**			**Episiotomy (n = 391)**		
Mean (SD)	26	(5.7)	Yes	208	53.5
≤29	457	71.5	No	181	46.5
30-39	171	26.8	**Birthweight (n = 628) (g)**		
≥40	11	1.7	<2500	53	8.2
**Parity (n = 638)**			2500-3999	562	86.7
Primiparous	493	74.9	≥4000	33	5.1
Multiparous	165	25.1	**Adequacy of birthweight (n = 627)**		
**Maternal anemia (n = 590)**			SGA	36	5.6
Yes	93	15.3	AGA	539	83.3
No	468	76.7	LGA	72	11.1
Missing	49	8.0	**APGAR 1^st^ minute (n = 623)**		
**Marital Status (n = 619)**			< 7	62	9.6
With partner	465	72.8	≥ 7	581	90.4
Without partner	174	27.2	**APGAR 5^th^ minute (n = 623)**		
**Gestational age at first prenatal visit (weeks)**			< 7	2	0.3
up to 5	23	3.5	≥ 7	641	99.7
6 to 12	482	72.9	**Somatic neonatal age (n = 606) (weeks)**		
12 to 16	156	23.6	< 37	51	8.2
Mean (SD)	9.9	(2.8)	≥ 37	575	91.9
**Initial BMI (kg/m^2^)**					
Low weight (<18.5)	36	5.5			
Adequate weight (18.5-24.99)	311	47.1			
Overweight (25-29.99)	204	30.9			
Obese (≥30)	110	16.9			
Mean (SD)	25.3	(5.2)			

SD(standart desviation),SGA (small for gestational age), AGA (adequate for gestational age), LGA (large for gestational age).

**Table 2:** Gestational weight gain according to initial BMI and comparison with the IOM recommendations.

WHO BMI classification at first prenatal visit			BMI increase in gestation*				Weight gain in gestation*					
	N	%	mean	SD	%		mean	SD	IOM recommendation		% of extra IOM**	p-value
					mean	SD						
**Low weight**	36	5.5	4.6	1.8	26.1	10.0	12.1	4.7	12.5	18.0	11.8	0.6324
**Adequate weight**	311	47.1	4.4	1.9	20.3	9.0	11.5	5.0	11.5	16.0	15.7	0.6695
**Overweight**	204	46.1	3.9	2.5	14.5	9.5	10.1	6.4	7.0	11.5	38.2	< 0.0001
**Obesity**	110	16.6	2.7	2.2	8.2	6.5	7.2	5.8	5.0	9.0	33.7	< 0.0001

* The initial weight was considered the weight at the first prenatal care visit (max: 12 weeks).

** Percentage of women with weight gain greater than the IOM recommendation, according to the original BMI classification.

WHO = World Health Organization.

BMI = body mass index.

SD = standard deviation.

IOM = Institute of Medicine.

*p*-value = T test of mean weight gain during gestation and respective mean of IOM recommendation.

**Table 3:** Equations for generation of smoothed curves of BMI percentiles during pregnancy for all women and according to initial BMI.

Weight Gain	Percentile		Intercept		Slope X GA		R^2^ of model	BMI (kg/m^2^) of percentile example for GA (weeks)		
								12	20	28
**Any Initial BMI**										
**Adequate Gain**	P 5	=	16.8	+	0.147	X GA	0.90	18.6	19.7	20.9
	P 10	=	17.4	+	0.168	X GA	0.96	19.4	20.8	22.1
	P 50	=	22.9	+	0.155	X GA	0.93	24.8	26.0	27.2
	P 85	=	28.8	+	0.151	X GA	0.79	30.6	31.8	33.0
**Excessive Gain**	P 90	=	31.3	+	0.134	X GA	0.62	32.9	34.0	35.1
	P 95	=	34.3	+	0.122	X GA	0.80	35.8	36.7	37.7
**Low Weight (Initial BMI <18.5 kg/m^2^)**										
**Adequate Gain**	P 5	=	15.1	+	0.110	X GA	0.57	16.4	17.3	18.2
	P 10	=	14.8	+	0.133	X GA	0.73	16.4	17.5	18.5
	P 50	=	16.2	+	0.156	X GA	0.93	18.1	19.3	20.6
	P 85	=	16.1	+	0.212	X GA	0.93	18.6	20.3	22.0
**Excessive Gain**	P 90	=	16.5	+	0.207	X GA	0.85	19.0	20.6	22.3
	P 95	=	16.1	+	0.240	X GA	0.83	19.0	20.9	22.8
**Adequate Weight (18.5 ≤ Initial BMI <25 kg/m^2^)**										
**Adequate gain**	P 5	=	17.2	+	0.145	X GA	0.93	18.9	20.1	21.3
	P 10	=	17.4	+	0.163	X GA	0.96	19.4	20.7	22.0
	P 50	=	20.2	+	0.167	X GA	0.93	22.2	23.5	24.9
	P 85	=	22.1	+	0.178	X GA	0.94	24.2	25.7	27.1
**Excessive Gain**	P 90	=	22.3	+	0.190	X GA	0.95	24.6	26.1	27.6
	P 95	=	22.6	+	0.207	X GA	0.95	25.1	26.7	28.4
**Overweight (25 ≤ Initial BMI <30 kg/m^2^)**										
**Adequate Gain**	P 5	=	23.8	+	0.090	X GA	0.85	24.9	25.6	26.3
	P 10	=	24.0	+	0.106	X GA	0.92	25.3	26.1	27.0
	P 50	=	24.9	+	0.159	X GA	0.94	26.8	28.1	29.4
	P 85	=	26.6	+	0.183	X GA	0.93	28.8	30.3	31.7
**Excessive Gain**	P 90	=	27.0	+	0.186	X GA	0.94	29.2	30.7	32.2
	P 95	=	27.0	+	0.214	X GA	0.93	29.6	31.3	33.0
**Obese (Initial BMI ≥30 kg/m^2^)**										
**Adequate Gain**	P 5	=	30.0	+	0.040	X GA	0.35	30.5	30.8	31.1
	P 10	=	30.3	+	0.070	X GA	0.55	31.1	31.7	32.3
	P 50	=	32.8	+	0.115	X GA	0.72	34.2	35.1	36.0
	P 85	=	37.9	+	0.070	X GA	0.14	38.7	39.3	39.9
**Excessive Gain**	P 90	=	39.0	+	0.061	X GA	0.09	39.7	40.2	40.7
	P 95	=	40.6	+	0.079	X GA	0.12	41.5	42.2	42.8

GA = gestational age, BMI = body mass index.

P = percentile, R^2^ = determination coefficient.

Weight gain: underweight = any BMI value lower than P5; Obesity = any BMI value greater than P95.

**Table 4:** Reference values of BMI according to initial BMI classification: low-weight and adequate-weight pregnant women.

	Low Weight (Initial BMI <18.5 kg/m^2^)					Adequate Weight (18.5≤ Initial BMI <25 kg/m^2^)					Overweight (25≤ Initial BMI <30 kg/m^2^)					Obese (Initial BMI ≥30 kg/m^2^)				
GA	n	Mean	SD	P5%	P95%	n	Mean	SD	P5%	P95%	n	Mean	SD	P5%	P95%	n	Mean	SD	P5%	P95%
**<8**	9	17.7	0.53	15.9	17.8	88	22.1	1.77	18.3	24.0	55	26.3	2.18	24.4	28.5	26	35.2	4.14	30.3	41.2
**8**	7	17.7	0.96	16.0	18.0	56	22.0	1.90	18.4	24.2	42	27.1	1.37	24.5	28.7	11	35.4	3.86	30.4	41.3
**9**	5	17.5	1.02	16.1	18.2	41	21.8	2.07	18.5	24.4	27	27.4	1.66	24.6	28.9	21	34.1	3.37	30.4	41.4
**10**	8	17.2	1.15	16.2	18.5	55	22.1	1.88	18.7	24.6	35	26.7	1.51	24.7	29.1	23	35.0	3.42	30.4	41.4
**11**	6	18.1	0.68	16.3	18.7	55	22.0	1.91	18.8	24.8	28	27.1	1.68	24.8	29.3	18	34.3	3.38	30.5	41.5
**12**	7	17.8	1.31	16.4	19.0	58	22.2	1.97	19.0	25.0	46	26.9	1.58	24.9	29.6	19	35.0	4.98	30.5	41.6
**13**	5	17.8	1.05	16.5	19.2	58	21.8	1.76	19.1	25.2	45	27.3	1.59	25.0	29.8	18	35.3	2.83	30.6	41.7
**14**	7	18.7	1.77	16.7	19.4	53	22.2	2.16	19.3	25.4	50	27.0	1.58	25.1	30.0	26	34.1	3.49	30.6	41.8
**15**	14	17.7	1.13	16.8	19.7	76	22.1	1.93	19.4	25.6	49	26.9	1.82	25.2	30.2	33	34.8	3.51	30.7	41.8
**16**	6	18.5	0.37	16.9	19.9	47	22.4	2.31	19.6	25.9	37	27.2	1.73	25.3	30.4	22	34.1	2.94	30.7	41.9
**17**	4	18.1	0.97	17.0	20.2	67	22.6	1.67	19.7	26.1	37	27.4	1.46	25.4	30.6	21	35.2	4.63	30.8	42.0
**18**	6	19.0	1.36	17.1	20.4	56	23.0	1.87	19.9	26.3	43	28.0	1.76	25.5	30.8	14	35.5	2.61	30.8	42.1
**19**	7	19.0	0.65	17.2	20.6	62	23.1	1.93	20.0	26.5	54	27.6	1.78	25.6	31.1	21	35.8	3.97	30.8	42.1
**20**	11	18.6	1.12	17.3	20.9	61	23.0	2.23	20.1	26.7	34	27.9	1.61	25.7	31.3	27	34.7	2.90	30.9	42.2
**21**	7	18.6	1.50	17.4	21.1	74	23.4	2.11	20.3	26.9	38	28.7	2.01	25.8	31.5	25	34.4	2.55	30.9	42.3
**22**	8	19.6	1.28	17.5	21.4	55	23.9	2.08	20.4	27.1	48	28.4	1.81	25.8	31.7	18	37.9	4.48	31.0	42.4
**23**	4	18.8	1.44	17.6	21.6	53	23.2	2.00	20.6	27.3	43	28.6	1.86	25.9	31.9	22	36.7	4.25	31.0	42.5
**24**	10	19.5	1.29	17.8	21.8	69	24.1	2.11	20.7	27.5	49	28.8	1.45	26.0	32.1	25	35.6	2.45	31.1	42.5
**25**	6	20.6	0.73	17.9	22.1	70	24.2	2.08	20.9	27.7	35	28.7	2.00	26.1	32.3	21	34.8	4.19	31.1	42.6
**26**	6	20.8	1.30	18.0	22.3	62	24.4	1.96	21.0	27.9	48	29.4	1.81	26.2	32.5	29	35.5	3.25	31.2	42.7
**27**	7	20.6	1.59	18.1	22.6	58	24.1	1.85	21.2	28.1	52	29.2	2.19	26.3	32.8	18	36.3	3.08	31.2	42.8
**28**	6	19.6	1.00	18.2	22.8	66	25.0	2.10	21.3	28.3	45	29.3	3.21	26.4	33.0	19	36.2	3.59	31.2	42.9
**29**	11	20.0	1.64	18.3	23.0	65	24.9	2.29	21.5	28.5	32	29.1	1.68	26.5	33.2	29	36.0	3.32	31.3	42.9
**30**	10	20.9	1.34	18.4	23.3	81	25.1	2.04	21.6	28.7	53	29.8	2.27	26.6	33.4	31	36.1	3.44	31.3	43.0
**31**	8	21.9	1.69	18.5	23.5	74	24.9	2.28	21.7	29.0	52	30.1	2.03	26.7	33.6	31	36.7	3.47	31.4	43.1
**32**	10	20.4	2.31	18.6	23.8	92	25.6	2.28	21.9	29.2	62	30.0	1.98	26.8	33.8	33	36.1	2.57	31.4	43.2
**33**	12	20.8	2.08	18.8	24.0	81	25.6	2.28	22.0	29.4	68	30.1	2.18	26.9	34.0	32	36.9	3.42	31.5	43.2
**34**	13	21.3	1.40	18.9	24.2	104	26.0	2.17	22.2	29.6	62	30.2	2.24	27.0	34.3	32	36.4	2.96	31.5	43.3
**35**	13	22.4	1.70	19.0	24.5	122	26.0	2.16	22.3	29.8	79	30.7	2.30	27.1	34.5	38	37.4	3.51	31.6	43.4
**36**	15	21.7	1.63	19.1	24.7	137	26.4	2.50	22.5	30.0	107	30.7	2.22	27.2	34.7	54	37.5	3.69	31.6	43.5
**37**	28	22.1	1.86	19.2	25.0	165	26.6	2.33	22.6	30.2	125	31.2	3.01	27.3	34.9	57	37.5	3.45	31.6	43.6
**38**	25	22.3	2.00	19.3	25.2	172	26.6	2.24	22.8	30.4	99	31.1	2.48	27.4	35.1	53	37.4	3.21	31.7	43.6
**39**	11	23.2	2.17	19.4	25.4	119	26.6	2.18	22.9	30.6	86	31.2	2.75	27.5	35.3	49	36.1	3.30	31.7	43.7
**40**	5	23.1	2.35	19.5	25.7	45	27.2	2.43	23.1	30.8	38	31.0	2.52	27.6	35.5	17	36.6	3.79	31.8	43.8

P5% - 5th Percentile - Inferior Limit; P95% - 95th Percentile - Superior Limit.

**Table 5:** Concordance between BMI classifications during pregnancy by Atalah’s curve and the proposed new curve (for all women).

Curves	Beginning of prenatal period		Middle (19 - 23 weeks)		Last prenatal visit	
Atalah’s / Proposed Curve	n	%	n	%	n	%
**Concordant**						
Low Weight	26	4.2	37	5.6	30	4.5
Adequate Weight	252	40.6	267	40.3	233	35.2
Overweight	0	0.0	0	0.0	0	0.0
Obesity	22	3.5	29	4.4	57	8.6
***TOTAL of concordant***	***300***	***48.3***	***333***	***50.2***	***320***	***48.3***
**Discordant**						
Adequate / Low Weight	3	0.5	9	1.4	50	7.6
Overweight / Adequate	172	27.7	171	25.8	177	26.7
Overweight / Obesity	71	11.4	67	10.1	45	6.8
Obesity / Overweight	75	12.1	83	12.5	70	10.6
Adequate / Overweight	0	0.0	0	0.0	0	0.0
Low Weight / Adequate	0	0.0	0	0.0	0	0.0
***TOTAL of discordant***	***321***	***51.7***	***330***	***49.8***	***342***	***51.7***
McNemar’s p-value	<0.0001		<0.0001		<0.0001	
kappa (CI)	0.36 (0.32-0.41)		0.39 (0.35-0.44)		0.37 (0.33-0.42)	

CI = confidence interval.
